# Ecophysiology of *Aspergillus* Section *Nigri* Species Potential Ochratoxin A Producers

**DOI:** 10.3390/toxins2112593

**Published:** 2010-10-29

**Authors:** Andrea L. Astoreca, Carina E. Magnoli, Ana M. Dalcero

**Affiliations:** Departamento de Microbiología e Inmunología, Facultad de Ciencias Exactas, Físico-Químicas y Naturales, Universidad Nacional de Río Cuarto, Ruta Nacional Nº 36 Km 601, (5800) Río Cuarto, Córdoba, Argentina; Email: cmagnoli@exa.unrc.edu.ar (C.M); adalcero@exa.unrc.edu.ar (A.D.)

**Keywords:** *Aspergilus niger* aggregate, *Aspergillus carbonarius*, ochratoxin A, water activity, temperature

## Abstract

After aflatoxins, ochratoxin A (OTA) is the most studied mycotoxin due to the toxicological significance in human and animal diets. OTA presence has been extensively reported worldwide in the last decade in several agricultural products. The main OTA producer in tropical and temperate climates is *Aspergillus carbonarius* followed by species belonging to *A. niger* aggregate. Currently, many scientists worldwide have studied the influence of water activity and temperature for growth and biosynthesis of OTA by these species on synthetic media. This article reviews ecophysiological studies of *Aspergillus* section *Nigri* strains on synthetic media and natural substrates. The results of these investigations suggest that significant amounts of OTA can be produced in only five days and that the use of different storage practices, such as *a*_W_ and temperature levels below 0.930 and 15 °C, respectively, allow controlling fungal contamination and minimizing the OTA production in several products as peanuts, corn, dried grapes and derived products for human consumption.

## 1. Introduction

The *Aspergillus* genus is distributed worldwide but is most commonly isolated from latitudes 26°–35° north or south of the Ecuador. Therefore, these fungi are common in warm and temperate climates. Their ability to develop under conditions of high temperature and relatively low *a*_w_ allows them to adapt and colonize a diverse array of cereals and dried fruits [1].

Some of the species belonging to this genus, *A. niger*, *A. sojae* and *A. oryzae*, are known as producers of industrial enzymes and metabolites able to give flavor to food. However, there are other species capable to infect plant tissue and produce mycotoxins [2]. Ochratoxins—the second mycotoxin group in importance after aflatoxins—includes at least nine metabolites that are similar in structural terms, of which ochratoxin A (OTA) is the most studied metabolite due to its occurrence in food and feed and toxicological significance in human and animal diets. This toxin is known to have nephrotoxic, immunotoxic, teratogenic and carcinogenic effects on animals. The International Agency for Research of Cancer (IARC) has classified OTA as a group 2B carcinogen based on toxicity on rats [3,4]. For these reasons, the European Commission has fixed maximum limits for OTA in several agricultural products destined for human and animal consumption [5].

OTA presence has been extensively reported worldwide in the last decade in several products, such as coffee [6,7], wines [8,9,10,11,12,13,14], beers [15,16,17], grapes [18,19,20], dried grapes [7,21,22,23,24], cereals and derivatives destined for humans and animals [25,26,27,28,29,30,31,32,33,34], oilseeds and derivates products [35] and occasionally, in body fluids, plasm, meat and kidneys of several animal species [36,37]. 

In the 90s, this toxin was considered to be only produced by *Penicillium verrucosum* in temperate and cold climates, and *A. ochraceus* and related species in warm and tropical climates. Due to their physiological differences, each of these species occupies a particular ecological niche. In the last years, several studies reported the presence of potential OTA producers *Aspergillus* section *Nigri* species in food and feed; considering *Aspergillus carbonarius* as the main OTA producer followed by species belonging to the *A. niger* aggregate [38]. 

In Argentina, as in other temperate and tropical countries, *P. verrucosum* and *A. ochraceus* have not been reported as frequent colonizers in agricultural products, while *A. carbonarius*, *A. niger* aggregate species and monoseriates *Aspergillus* species such as *A. aculeatus* and *A. japonicus* have been frequently isolated from several agricultural products, e.g., coffee beans, red wine, dried grapes, corn and peanut kernels and feeds. OTA was detected in some of these substrates except in coffee beans and corn kernels [10,13,21,25,35,39,40,41,42,43]. In others South American countries, several authors have also informed the presence of this species in cocoa and coffee beans, grapes and poultry feed [44,45,46,47,48].

Crops contaminated with mycotoxins reflect a loss of income for agricultural producers. The strategies that prevent the entry of these metabolites in the food chain protect public health and reduce economic losses caused by contaminated agricultural products. 

The knowledge of the ecophysiology of OTA producing fungi and ecological factors that influence OTA production is essential to optimize the implementation of preventive strategies aimed at controlling the sanitary quality of raw materials and/or products susceptible to fungal colonization.

There are multiple factors significantly involved in the development of section *Nigri* species and secondary metabolites biosynthesis, e.g., humidity, temperature, presence of oxygen and carbon dioxide, incubation time, substrate composition, loss of grain integrity caused by insects or mechanical/thermal damage, fungal inoculum, and the interaction/competition between other contaminated fungal species. Of these factors, water activity (*a*_W_) and temperature have shown the greatest effects on growth and OTA production. In general, the toxigenic species are not aggressive pathogens, but are often well adapted to substrates with low humidity, and they can easily colonize cereal grains and oilseeds that are stored under inappropriate environmental conditions.

In recent years, scientific studies have focused mainly on the influence of environmental factors on OTA production by *P. verrucosum* and *A. ochraceus* since they are recognized as the main producer species. Several ecophysiological studies on *A. ochraceus* were reported in the two last decades [49,50,51,52,53,54,55] as well as studies on *P. viridicatum*, nowadays classified as *P. verrucosum* [56,57,58,59].

Currently, many scientists worldwide have studied the influence of water activity and temperature for biosynthesis of OTA by other ochratoxigenic species belonging to section *Nigri* such as *A. niger* aggregate and *A. carbonarius* [60,61,62,63,64,65,66,67,68,69,70,71,72,73,74,75,76,77,78,79], although only a few studies have reported the effect of incubation time on the amount of OTA produced by some strains belonging to the genera *Aspergillus* section *Nigri* [80,81,82].

Besides environmental factors, biological factors have a noticeable influence on growth and OTA production. Some of these biological factors are intrinsic; hence, depend only on the genetic basis of the fungal strain. The strains vary in their ability to produce OTA as well as the quantity produced. Recently, several studies have allowed the detection of some genes that may be involved in the biosynthesis of this toxin. Initially, genes encoding a polyketide synthase of *A. ochraceus* [83,84], *P. verrucosum* [85] and *P. nordicum* [86] were characterized; but these genes had low homology between the last two species. The simultaneous presence of different bacteria or other fungi in the substrates also influences the growth of these ochratoxigenic species and OTA production. 

## 2. Ecophysiological Studies of *Aspergillus* Section *Nigri* Strains on Synthetic Media

Several authors have shown minima and optima conditions for mycelial growth of ochratoxigenic species belonging to *Aspergillus* section *Nigri* on different culture media [60,64,65,66,67,68,69,70,71,72,76,77,79,80,82] (Table 1). In these studies, growth rates of *Aspergillus* section *Nigri* species showed a marked decrease with the reduction of temperature and water activity level, and were not higher than 3 mm day^−^^1^ at 15 °C at the lowest *a*_W_. The highest growth rates of these species found at optimal conditions differ depending on the agar media used. The highest growth rates achieved by *A. carbonarius* species was found on CYA by Romero *et al.* [79] at 0.95 *a*_W_ and 30 °C (17.46 mm day^−1^) with a strain isolated from Argentinean dried vine fruits and Leong *et al.* [77] reported a maximum growth rate (11.35 mm day^−1^) for an *A. niger* aggregate strain at 0.98 *a*_W_ and 35 °C on a simulated grape juice medium. However, the literature shows that in most combinations of temperature and aw tested, *A. niger* grew more rapidly than *A. carbonarius*. The reproducibility of these optima for growth indicates that *A. niger* aggregate has a higher optimum temperature for growth than *A. carbonarius* and possibly also takes greater advantage of high water activities for rapid growth. In general, the optimum conditions for growth of assayed strains belonging to the *A. niger* aggregate and *A. carbonarius* strains were similar than that reported by the former authors. These optimal conditions for growth by *Aspergillus* section *Nigri* strains are in agreement with previous European works [60,63,70,81], which reported optimal growth rates for *A. carbonarius* strains at temperatures between 30–35 °C and 0.950-0.980 *a*_W_. The environmental conditions of growth inhibition observed by Astoreca *et al.* [64] agree with those obtained previously on synthetic media with strains of section *Nigri* isolated from maize grains [87] and from grapes [60,70,81]. In another study from Argentina, Romero *et al.* [79] evaluated the growth rate of *A. carbonarius* strains isolated from dried grapes at different *a*_W_ and temperature conditions and the results showed that the inhibition of growth occurred at 15 °C among 0.825 to 0.900 *a*_W_, and 25 °C at 0.825 *a*_W_.

Ochratoxin A production in relation to ecophysiological factors by *Aspergillus* section *Nigri* strains was evaluated by several authors on synthetic media [62,63,65,66,67,68,69,70,71,73,81,82] (Table 2). 

Astoreca *et al.* [65] evaluated the influence of ecophysiological factors on OTA production by six *A. niger* aggregate strains isolated from peanut seeds, corn kernels and coffee beans; and two *A. carbonarius* strains isolated from dried grapes in Argentina. These authors observed that the optimum temperature for OTA production was 25 °C for all strains belonging to the *A. niger* aggregate; while the *A. carbonarius* strains produced the highest OTA levels at 25 and 30 °C, without significant differences in production profiles between these strains. These OTA production patterns agree with those previously reported by Bellí *et al.* [72]. In this study, the results of OTA production by *Aspergillus* section *Nigri* species on a medium similar to grape composition were modeled by a multiple linear regression and predictive models were obtained. The results showed that high levels of *a*_W_ (0.960 and 0.995) favored OTA production by *A. carbonarius* and *A. niger* aggregate strains as Bellí *et al.* [81], Esteban *et al.* [61,62], Joosten *et al.* [6] and Romero *et al.* [78] reported. On the other hand, Oueslati *et al.* [80] evaluated the effect of three alternating temperatures cycles (20/30, 20/37 and 25/42 °C) and photoperiod on growth and OTA production of six strains of *A. carbonarius* from Tunisian grapes on synthetic nutrient medium. The different temperature regimes assayed affected significantly both the mycelial growth and the OTA production. The best growth and OTA production were recorded with the 20/30 °C cycle.

Several studies have detected similar minimum *a*_W_ level, 0.90–0.94 for OTA production by *Aspergillus niger* aggregate and *A. carbonarius* strains on different synthetic media (Table 2), whereas, Romero *et al.* [78] reported *A. carbonarius* strains able to produce traces amounts of OTA at lowest *a*_W_ level (0.87) on CYA medium. The results present in the literature shows that different substrates significantly affect the optimum and the minimum *a*_W_ conditions for OTA synthesis.

## 3. Ecophysiological Studies of *Aspergillus* Section *Nigri* Strains on Natural Substrates

Results obtained on synthetic substrates may not accurately represent the real fungal ability to produce OTA on a natural substrate. Therefore, studies on OTA production have been frequently carried out on natural and common substrates for ochratoxigenic *A. ochraceus* and *P. verrucosum*, such as cereal grains, green coffee beans and grapes, but there is little information about the influence of *a*_W_ and temperature on growth parameters and OTA production of *Aspergillus* section *Nigri* strains on natural substrates. 

**Table 1:** Optima and minima conditions (temperature and water activity) for *Aspergillus* section *Nigri* species growth on different culture media.

Fungal species	Medium	Tested temperature range (°C)	Optimum temperature (°C)	Optimum *a*_W_	Minimum *a*_W_	References
*A.* section *Nigri*	Grains and fruits based	15–30	30	0.97	0.85	Astoreca *et al.* [64]
	Synthetic nutrient	10–37	30–37	0.98	-	Bellí *et al.* [72]
	CYA-YES	5–45	30–35	-	-	Esteban *et al.* [82]
*A. niger* aggregate	Corn grains	15–30	30	0.95	0.91	Astoreca *et al.* [66]
	Peanut seeds	15–30	30	0.99	0.91	Astoreca *et al.* [67]
	Coffee beans	15–30	25	0.99	0.93	Astoreca *et al.* [68]
	Synthetic nutrient	10–37	25	0.95	-	Selouane *et al.* [76]
	Simulated grape juice	15–35	35	0.98	0.92 (15 °C)	Leong *et al.* [77]
*A. carbonarius*	Synthetic grape juice	10–40	35	0.98	0.88	Mitchell *et al.* [60]
	Dried grapes	15–30	25–30	0.99	0.91	Astoreca *et al.* [64]
	Synthetic grape	15–37	30	0.95–0.99	0.90 (15 °C)	Bellí *et al.* [70]
	Synthetic nutrient	20–28	28	-	-	Bellí *et al.* [71]
	Synthetic nutrient	10–37	25	0.95	-	Selouane *et al.* [76]
	Simulated grape juice	15–35	30	0.965	0.92 (15 °C)	Leong *et al.* [77]
	CYA	15–35	30	0.95	0.85	Romero *et al.* [79]
	Synthetic nutrient	20/30	20/30	-	-	Oueslati *et al.* [80]
		20/37				
		25/42				

CYA: Czapek Yeast Autolysate agar; YES: Yeast Extract Sucrose agar; -: Data not reported.

**Table 2:** Optima and minima conditions for ochratoxin A (OTA) production by *Aspergillus* section *Nigri* species on different culture media.

Fungal species	Medium	Tested temperature range (°C)	Optimum temperature (°C)	Optimum *a*_W_	Minimum *a*_W_	References
*A.* section *Nigri*	Grains and fruits based	15–30	25–30	0.97–0.99	0.89–0.90	Astoreca *et al.* [65]
	CYA-YES	5–45	25	-	-	Esteban *et al.* [82]
*A. niger* aggregate	CYA-YES	-	-	0.99	0.94	Esteban *et al.* [61]
		15–30	15	0.98	0.90	Esteban *et al.* [62]
	Corn grains	15–30	25	0.97	0.91–0.93	Astoreca *et al.* [66]
	Peanut seeds	15–30	25	0.97 (25°C)	0.91	Astoreca *et al.* [67]
	Coffee beans	15–30	30	0.99	0.95	Astoreca *et al.* [68]
	Synthetic nutrient	10–37	30–37	0.90–0.95	-	Selouane *et al.* [76]
	Simulated grape juice	15–35	15	0.95	-	Leong *et al.* [77]
	Synthetic nutrient	10–37	30	0.995	-	Bellí *et al.* [81]
*A. carbonarius*	Coffee cherries	15–35	25	0.99	0.94	Joosten *et al.* [6]
	Synthetic grape juice	10–40	15–20	0.95–0.98	-	Mitchell *et al.* [60]
	Dried grapes	15–30	30	0.99	0.91	Astoreca *et al.* [69]
	Synthetic grape	15–37	20	0.95–0.99	0.90	Bellí *et al.* [70]
	Synthetic Nutrient	20–28	28	-	-	Bellí *et al.* [71]
	Table grapes	20–30	30	0.99	-	Bellí *et al.* [73]
	Synthetic nutrient	10–37	25–30	0.95–0.99	-	Selouane *et al.* [76]
	Simulated grape juice	15–35	15	0.95–0.98	-	Leong *et al.* [77]
	CYA	15–35	15	0.95	0.87	Romero *et al.* [78]
	Synthetic nutrient	20/30	20/30	-	-	Oueslati *et al.* [80]
		20/37				
		25/42				
	Synthetic nutrient	10–37	25	0.96	-	Bellí *et al.* [81]

CYA: Czapek Yeast Autolysate agar; YES: Yeast Extract Sucrose agar; -: Data not reported.

Most of the studies were done on solid synthetic and different substrate based media. The first study carried out with OTA producing strains belonging to *Aspergillus* section *Nigri* on natural substrate was reported by Joosten *et al.* [6], who studied the effect of environmental factors on growth parameters and OTA production by *A. carbonarius* strains on coffee cherries in Thailand. These authors showed that the optimal conditions of OTA production by *A. carbonarius* were 25 °C and 0.990 *a*_W_. In other study, four ochratoxigenic *A. carbonarius* strains isolated from wine grapes were used to inoculate artificially damaged and undamaged table grapes [73]. Grapes were stored at three levels of relative humidity (80, 90 and 100%) and at two temperatures (20 and 30 °C). The results showed that temperature and relative humidity significantly influenced both infection and toxin content. At 30 °C, the detected OTA amount was higher than at 20 °C in most of the treatments. The highest relative humidity (100%) led to maximum OTA amounts while no significant differences were found between 90% and 80% in the OTA content. 

Recently, Astoreca *et al.* [66,67,68,69] reported on the influence of environmental factors (*a*_W_: 0.995, 0.973, 0.951, 0.928 and 0.910 and temperature: 15, 25 and 30 °C) on lag phase, growth rate, and OTA production by strains belonging to *A. niger* aggregate on irradiated corn, peanuts, dried grapes and coffee beans at 7, 14 and 21 days of incubation. The observed general pattern of *in vitro* growth [64,65] was different from those obtained on the natural substrates. The assayed strains on irradiated peanut seeds showed the shortest lag phases at the optimum conditions (7 h at 30 °C and 0.995) while the other strains reached the exponential phases only after around 35.3, 13.2 and 94.8 h at the same condition on irradiated corn, dried grapes and coffee beans, respectively. Under optimal conditions (0.973 *a*_W_, 25 °C and seven days of incubation), the OTA production in irradiated corn, peanut and dried grapes were significantly greater than the concentrations obtained by these strains on the medium based on each substrate. 

Marín *et al.* [88] developed a suitable validated model to predict the growth and OTA production boundaries by an *A. carbonarius* strain in the function of moisture content and storage temperature of pistachios. These authors showed that OTA accumulation was mainly a function of the temperature of storage, with a sharp increase at <15-20 °C; this value was very different from 30 to 35 °C, which was optimum for growth. This suggests that the substrate combined with certain environmental conditions on which strains are developed affects OTA production. According to these results, significant amounts of OTA on natural substrates could be produced in only five days at *a*_W_ ≥ 0.970 in a wide temperature range. These studies of predictive models represent an essential improvement to the quality and safety of food, allowing the prediction of toxin contamination.

## 4. Conclusions

In general, temperature and *a*_W_ range for OTA production is more restricted than fungal growth, both on natural substrate but also on the culture media based substrate. The knowledge of the ecophysiology of strains belonging to the genera *Aspergillus* section *Nigri* is critical in the development and prediction of risk models for contamination of both raw materials and finished foods with these species under the interaction of several environmental parameters. The use of different storage practices, such as *a*_W_ and temperature levels below 0.930 and 15 °C, respectively, allow controlling fungal contamination and minimizing OTA production in several products as peanuts, corn, dried grapes and derived products for human consumption.

## References

[1] Pitt J.I., Hocking A.D. (1997). Fungi and Food Spoilage.

[2] Perrone G., Susca A., Cozzi G., Ehrlich K., Varga J., Frisvad J.C., Meijer M., Noonim P., Mahakarnchanakul W., Samson R.A. (2007). Biodiversity of *Aspergillus* species in some important agricultural products. Stud. Mycol..

[3] International Agency for Research on Cancer (1993). Ochratoxin A. IARC Monographs on the Evaluation of Carcinogenic Risks to Human: Some Naturally Occurring Substances; Food Items and Constituents, Heterocyclic Aromatic Amines and Mycotoxins.

[4] JECFA (2001). Safety Evaluation of Certain Mycotoxins in Food.

[5] European Commission (2010). Commission Regulation (EU) No 105/2010 of 5 February 2010 amending Regulation (EC) No 1881/2006 setting maximum levels for certain contaminants in foodstuffs as regards ochratoxin A. Off. J. Eur. Union.

[6] Joosten H.M., Goetz J., Pittet A., Schellenberg M., Bucheli P. (2001). Production of ochratoxin A by *Aspergillus carbonarius* on coffee cherries. Int. J. Food Microbiol..

[7] Aoyama K., Nakajima M., Tabata S., Ishikuro E., Tanaka T., Norizuki H., Itoh Y., Fujita K., Kai S., Tsutsumi T. (2010). Four-year surveillance for ochratoxin A and fumonisins in retail foods in Japan. J. Food Prot..

[8] Ratola N., Barros P., Simoes T., Cerdeira A., Venancio A., Alves A. (2006). Worldwide interlaboratory study on the determination of ochratoxin A in different wine type samples. Talanta.

[9] Serra R., Mendonça C., Venancio A. (2006). Ochratoxin A occurrence and formation in Portuguese wine grapes at various stages of maturation. Int. J. Food Microbiol..

[10] Ponsone M.L., Combina M., Dalcero A., Chulze S. (2007). Ochratoxin A and ochratoxigenic *Aspergillus* species in Argentinean wine grapes cultivated under organic and non-organic systems. Int. J. Food Microbiol..

[11] Var I., Kabak B. (2007). Occurrence of ochratoxin A in Turkish wines. Microchem. J..

[12] Valero A., Marín S., Ramos A.J., Sanchis V. (2008). Survey: Ochratoxin A in European special wines. Food Chem..

[13] Ponsone M.L., Chiotta M.L., Combina M., Dalcero A.M., Chulze S.N. (2009). Fate of ochratoxin A content in Argentinian red wine during a pilot scale vinification. Rev. Argent. Microbiol..

[14] Tessini C., Mardones C., Von Baer D., Vega M., Herlitz E., Saelzer R., Silva J., Torres O. (2010). Alternatives for sample pre-treatment and HPLC determination of ochratoxin A in red wine using fluorescence detection. Anal. Chim. Acta.

[15] Medina A., Jiménez M., Gimeno-Adelantado J., Valle-Algarra F., Mateo R. (2005). Determination of ochratoxin A in beer marketed in Spain by liquid chromatography with fluorescence detection using lead hydroxyacetate as a clean-up agent. J. Chromatogr. A.

[16] Mateo R., Medina A., Mateo E.M., Mateo F., Jiménez M. (2007). An overview of ochratoxin A in beer and wine. Int. J. Food Microbiol..

[17] Reinsch M., Topfer A., Lehmann A., Nehls I., Panne U. (2007). Determination of ochratoxin A in beer by LC-MS/MS ion trap detection. Food Chem..

[18] Battilani P., Magan N., Logrieco A. (2006). European research on ochratoxin A in grapes and wine. Int. J. Food Microbiol..

[19] Lasram S., Bellí N., Chebil S., Nahla Z., Ahmed M., Sanchís V., Ghorbel A. (2007). Short communication: Occurrence of ochratoxigenic fungi and ochratoxin A in grapes from a Tunisian vineyard. Int. J. Food Microbiol..

[20] Varga J., Kozakiewicz Z. (2006). Ochratoxin A in grapes and grape derived products. Trends Food Sci. Technol..

[21] Magnoli C., Astoreca A., Ponsone L., Combina M., Palacio G., Rosa C.A.R., Dalcero A. (2004). Survey of mycoflora and ochratoxin A in dried vine fruits from Argentina markets. Lett. Appl. Microbiol..

[22] Chulze S.N., Magnoli C.E., Dalcero A.M. (2006). Review: Occurrence of ochratoxin A in wine and ochratoxigenic mycoflora in grapes and dried vine fruits in South America. Int. J. Food Microbiol..

[23] Karbancıoglu-Guler F., Heperkan D. (2008). Natural occurrence of ochratoxin A in dried figs. Anal. Chim. Acta.

[24] Bircan C. (2009). Incidence of ochratoxin A in dried fruits and co-occurrence with aflatoxins in dried figs. Food Chem. Toxicol..

[25] Dalcero A., Magnoli C., Hallak C., Chiacchiera S.M., Palacio G., Rosa C.A.R. (2002). Detection of ochratoxin A in animal feeds and capacity to produce this mycotoxin by *Aspergillus* section *Nigri* in Argentina. Food Addit. Contam..

[26] Araguás C., González-Peñas E., López de Cerain A. (2005). Study on ochratoxin A in cereal-derived products from Spain. Food Chem..

[27] Magnoli C., Astoreca A., Chiacchiera S.M., Dalcero A. (2007). Occurrence of ochratoxin A and ochratoxigenic mycoflora in corn and corn based foods and feeds in some South American countries. Mycopathologia.

[28] Juan C., Moltó J.C., Lino C.M., Mañes J. (2008). Analytical methods: Determination of ochratoxin A in organic and non-organic cereals and cereal products from Spain and Portugal. Food Chem..

[29] Juan C., Pena A., Lino C., Moltó J.C., Mañes J. (2008). Levels of ochratoxin A in wheat and maize bread from the central zone of Portugal. Int. J. Food Microbiol..

[30] Juan C., Zinedine A., Idrissi L., Mañes J. (2008). Ochratoxin A in rice on the Moroccan retail market. Int. J. Food Microbiol..

[31] Villa P., Markaki P. (2009). Aflatoxin B_1_ and ochratoxin A in breakfast cereals from at hens market: Occurrence and risk assessment. Food Control.

[32] Zaied C., Abid S., Zorgui L., Bouaziz C., Chouchane S., Jomaa M., Bacha H. (2009). Natural occurrence of ochratoxin A in Tunisian cereals. Food Control.

[33] Duarte S.C., Pena A., Lino C.M. (2010). A review on ochratoxin A occurrence and effects of processing of cereal and cereal derived food products. Food Microbiol..

[34] Monbaliu S., Van Poucke C., Detavernier C., Dumoulin F., Van de Velde M., Schoeters E., Van Dyck F., Averkieva O., Van Peteghem C., De Saeger S. (2010). Occurrence of mycotoxins in feed as analyzed by a multi-mycotoxin LC-MS/MS method. J. Agric. Food Chem..

[35] Magnoli C., Astoreca A., Ponsone M.L., Fernández-Juri M.G., Barberis C., Dalcero A. (2007). Ochratoxin A and *Aspergillus* section *Nigri* in peanut seeds at different months of storage in Córdoba, Argentina. Int. J. Food Microbiol..

[36] Matrella R., Monaci L., Milillo M.A., Palmesano F., Tantillo M.G. (2006). Ochratoxin A determination in paired kidneys and muscle samples from swines slaughtered in southern Italy. Food Control.

[37] Aoudia N., Tangni E.K., Larondelle Y. (2008). Distribution of ochratoxin A in plasma and tissues of rats fed a naturally contaminated diet amended with micronized wheat fibers: Effectiveness of mycotoxin sequestering activity. Food Chem. Toxicol..

[38] Abarca M.L., Accensi F., Cano J., Cabañes F.J. (2004). Taxonomy and significance of black Aspergilli. Antonie Van Leeuwenhoek.

[39] Magnoli C., Hallak C., Astoreca A., Ponsone L., Chiacchiera S.M., Palacio G., Dalcero A. (2005). Surveillance of toxigenic fungi and ochratoxin A in feedstuffs from Córdoba province, Argentina. Vet. Res. Commun..

[40] Magnoli C., Hallak C., Chiacchiera S.M., Dalcero A. (2006). Occurrence of ochratoxin A-producing fungi in commercial corn kernels in Argentina. Mycophatologia.

[41] Magnoli C., Astoreca A., Ponsone L, Chiacchiera S.M., Dalcero A. (2006). Ochratoxin A producing fungi in stored peanut seeds from Córdoba province, Argentina. J. Sci. Food Agric..

[42] Romero S.M., Comerio R.M., Larumbe G., Ritieni A., Vaamonde G., Fernández Pinto V. (2005). Toxigenic fungi isolated from dried vine fruits in Argentina. Int. J. Food Microbiol..

[43] Chiotta M.L., Ponsone M.L., Combina M., Torres A.M., Chulze S.N. (2009). *Aspergillus* section *Nigri* species isolated from different wine-grape growing regions in Argentina. Int. J. Food Microbiol..

[44] Batista L.R., Chalfoun S.M., Prado G., Schwan R.F., Wheals A.E. (2003). Toxigenic fungi associated with processed (green) coffee beans (*Coffea arabica* L.). Int. J. Food Microbiol..

[45] Rosa C.A.R., Ribeiro J.M.M., Fraga M.J., Gatti M., Cavaglieri L.R., Magnoli C.E., Dalcero A.M., Lopes C.W.G. (2006). Mycoflora of poultry feeds and ochratoxin-producing ability of isolated *Aspergillus* and *Penicillium* species. Vet. Microbiol..

[46] Sartori D., Furlaneto M.C., Martins M.K., Ferreira de Paula M.R., Pizzirani-Kleiner A.A., Taniwaki M.H., Pelegrinelli Fungaro M.H. (2006). PCR method for the detection of potential ochratoxin-producing *Aspergillus* species in coffee beans. Res. Microbiol..

[47] Díaz G.A., Torres R., Vega M., Latorre B.A. (2009). Ochratoxigenic *Aspergillus* species on grapes from Chilean vineyards and *Aspergillus* threshold levels on grapes. Int. J. Food Microbiol..

[48] Copetti M.V., Pereira J.L., Iamanaka B.T., Pitt J.I., Taniwaki M.H. (2010). Ochratoxigenic fungi and ochratoxin A in cocoa during farm processing. Int. J. Food Microbiol..

[49] Ramakrishna N., Lacey J., Smith J.E. (1996). Colonization of barley grain by *Penicillium verrucosum* and ochratoxin A formation in the presence of competing fungi. J. Food Prot..

[50] Lee H.B., Magan N. (1999). Environmental factors influence *in vitro* interespecific interactions between *A. ochraceus* and other maize spoilage fungi, growth and ochratoxin production. Mycophatologia.

[51] Ramos A.J., Labernia N., Marin S., Sanchís V., Magan N. (1998). Effect of water activity and temperature on growth and ochratoxin production by three strains of *Aspergillus ochraceus* on a barley extract medium and on barley grains. Int. J. Food Microbiol..

[52] Kapetanakou A.E., Panagou E.Z., Gialitaki M., Drosinos E.H., Skandamis P.N. (2009). Evaluating the combined effect of water activity, pH and temperature on ochratoxin A production by *Aspergillus ochraceus* and *Aspergillus carbonarius* on culture medium and Corinth raisins. Food Control.

[53] Pardo E., Marín S., Sanchís V., Ramos A.J. (2004). Prediction of fungal growth and ochratoxin A production by *Aspergillus ochraceus* on irradiated barley grain as influenced by temperature and water activity. Int. J. Food Microbiol..

[54] Pardo E., Marín S., Sanchís V., Ramos A.J. (2005). Impact of relative humidity and temperature on visible fungal growth and OTA production of ochratoxigenic *Aspergillus ochraceus* isolates on grapes. Food Microbiol..

[55] Pardo E., Ramos A.J., Sanchís V., Marín S. (2005). Modeling of the effects of water activity and growth of ochratoxigenic isolates of *Aspergillus ochraceus* on a green coffee-based medium. Int. J. Food Microbiol..

[56] Pardo E., Marín S., Ramos A.J., Sanchís V. (2006). Ecophysiology of ochratoxigenic *Aspergillus ochraceus* and *Penicillium verrucosum* isolates. Predictive models for fungal spoilage prevention—a review. Food Addit. Contam..

[57] Arroyo M., Aldred D., Magan N. (2005). Environmental factors and weak organic acid interactions have differential effects on control of growth and ochratoxin A production by *Penicillium verrucosum* isolates in bread. Int. J. Food Microbiol..

[58] Czaban J., Wróblewska B., Stochmal A., Janda B. (2006). Growth of *Penicillium verrucosum* and production of ochratoxin A on nonsterilized wheat grain incubated at different temperatures and water content. Pol. J. Microbiol..

[59] Aldred D., Cairns-Fuller V., Magan N. (2008). Environmental factors affect efficacy of some essential oils and resveratrol to control growth and ochratoxin A production by *Penicillium verrucosum* and *Aspergillus westerdijkiae* on wheat grain. J. Stored. Prod. Res..

[60] Mitchell D., Parra R., Aldred D., Magan N. (2004). Water and temperature relations of growth and ochratoxin A production by *Aspergillus carbonarius* strains from grapes in Europe and Israel. J. Appl. Microbiol..

[61] Esteban A., Abarca M.L., Bragulat M.R., Cabañes F.J. (2006). Study of the effect of water activity and temperature on ochratoxin A production by *Aspergillus carbonarius*. Food Microbiol..

[62] Esteban A., Abarca M.L., Bragulat M.R., Cabañes F.J. (2006). Effect of water activity on ochratoxin A production by *Aspergillus niger* aggregate species. Int. J. Food Microbiol..

[63] Marín S., Bellí N., Lasram S., Chebil S., Ramos A.J., Ghorbel A. (2006). Kinetics of ochratoxin A production and accumulation by *Aspergillus carbonarius* on synthetic grape medium at different temperature levels. J. Food Sci..

[64] Astoreca A., Magnoli C., Ramirez M.L., Combina M., Dalcero A. (2007). Water activity and temperature effects on growth of *Aspergillus niger*, *A. awamori* and *A. carbonarius* isolated from different substrates in Argentina. Int. J. Food Microbiol..

[65] Astoreca A., Magnoli C., Barberis C., Chiacchiera S.M., Combina M., Dalcero A. (2007). Ochratoxin A production in relation to ecophysiological factors by *Aspergillus* section *Nigri* strains isolated from different substrates in Argentina. Sci. Total Environ..

[66] Astoreca A., Barberis C., Magnoli C., Combina M., Dalcero A. (2009). Ecophysiological factors effect on growth rate, lag phase and ochratoxin A production by *Aspergillus niger* aggregate strains in irradiated peanut seeds. Int. J. Food Microbiol..

[67] Astoreca A., Magnoli C., Barberis C., Combina M., Chiacchiera S.M., Dalcero A. (2009). Influence of ecophysiological factors on growth, lag phase and ochratoxin A production by *Aspergillus niger* aggregate strains in corn grains. Int. J. Food Microbiol..

[68] Astoreca A., Magnoli C., Barberis C., Combina M., Dalcero A. (2010). Growth and ochratoxin A production by *Aspergillus niger* group strains in coffee beans in relation to environmental factors. World Mycotoxin J..

[69] Astoreca A., Magnoli C., Barberis C., Combina M., Dalcero A. (2010). *Aspergillus carbonarius* growth and ochratoxin A production in irradiated dried grapes under different water activity and temperature conditions. World Mycotoxin J..

[70] Bellí N., Ramos A.J., Coronas I., Sanchís V., Marín S. (2005). *Aspergillus carbonarius* growth and ochratoxin A production on a synthetic grape medium in relation to environmental factors. J. Appl. Microbiol..

[71] Bellí N., Ramos A.J., Sanchís V., Marín S. (2006). Effect of photoperiod and day-night temperatures simulating field conditions on growth and ochratoxin A production of *Aspergillus carbonarius* strains isolated from grapes. Food Microbiol..

[72] Bellí N., Marín S., Sanchís V., Ramos A.J. (2004). Influence of water activity and temperature on growth of isolates of *Aspergillus* section *Nigri* obtained from grapes. Int. J. Food Microbiol..

[73] Bellí N., Marín S., Coronas I., Sanchís V., Ramos A.J. (2007). Skin damage, high temperature and relative humidity as detrimental factors for *Aspergillus carbonarius* infection and ochratoxin A production in grapes. Food Control.

[74] Valero A., Farré J.R., Sanchís V., Ramos A.J., Marín S. (2006). Effects of fungal interaction on ochratoxin A production by *A. carbonarius* at different temperatures and water activity. Int. J. Food Microbiol..

[75] Valero A., Begum M., Hocking A.D., Marín S., Ramos A.J., Sanchís V. (2008). Mycelial growth and ochratoxin A production by *Aspergillus* section *Nigri* on simulated grape medium in modified atmospheres. J. Appl. Microbiol..

[76] Selouane A., Bouya D., Lebrihi A., Decock C., Bouseta A. (2009). Impact of some environmental factors on growth and production of ochratoxin A of/by *Aspergillus tubingensis*, *A. niger*, and *A. carbonarius* isolated from Moroccan grapes. J. Microbiol.

[77] Leong S.L., Hocking A.D., Scout E.S. (2006). Effect of temperature and water activity on growth and ochratoxin A production by Australian *Aspergillus carbonarius* and *A. niger* isolates on a simulated grape juice medium. Int. J. Food Microbiol..

[78] Romero S.M., Fernández Pinto V., Patriarca A., Vaamonde G. (2010). Ochratoxin A production by a mixed inoculum of *Aspergillus carbonarius* at different conditions of water activity and temperature. Int. J. Food Microbiol..

[79] Romero S.M., Patriarca A., Fernández Pinto V., Vaamonde G. (2007). Effect of water activity and temperature on growth of ochratoxigenic strains of *Aspergillus carbonarius* isolated from Argentinean dried vine fruits. Int. J. Food Microbiol..

[80] Oueslati S., Lasram S., Ramos A.J., Marin S., Mliki A., Sanchís V., Ghorbel A. (2010). Alternating temperatures and photoperiod effects on fungal growth and ochratoxin A production by *Aspergillus carbonarius* isolated from Tunisian grapes. Int. J. Food Microbiol..

[81] Bellí N., Ramos A.J., Sanchís V., Marín S. (2004). Incubation time and water activity effects on ochratoxin A production by *Aspergillus* section *Nigri* strains isolated from grapes. Lett. Appl. Microbiol..

[82] Esteban A., Abarca M.L., Bragulat M.R., Cabañes F.J. (2004). Effects of temperature and incubation time on production of ochratoxin A by black aspergilli. Res. Microbiol..

[83] O’Callaghan J., Caddick M.X., Dobson A.D.W. (2003). A polyketide synthase gene required for ochratoxin A biosynthesis in *Aspergillus ochraceus*. Microbiology.

[84] Abbas A., Valez H., Dobson A.D. (2009). Analysis of the effect of nutritional factors on OTA and OTB biosynthesis and polyketide synthase gene expression in *Aspergillus ochraceus*. Int. J. Food Microbiol..

[85] Schmidt-Heydt M., Baxter E., Geisen R., Magan N. (2007). Physiological relationship between food preservatives, environmental factors, ochratoxin and otapksPV gene expression by *Penicillium* *verrucosum*. Int. J. Food Microbiol..

[86] Geisen R. (2004). Molecular monitoring of environmental conditions influencing the induction of ochratoxin A biosynthesis genes in *Penicillium nordicum*. Mol. Nutr. Food Res..

[87] Marín S., Sanchís V., Ramos A.J., Vinas I., Magan N. (1998). Environmental factors, *in vitro* interactions, and niche overlap between *Fusarium moniliforme*, *F. proliferatum* and *F. graminearum*, *Aspergillus* and *Penicillium* species from maize grain. Mycol. Res..

[88] Marín S., Hodžić I., Ramos A.J., Sanchís V. (2008). Predicting the growth/no-growth boundary and ochratoxin A production by *Aspergillus carbonarius* in pistachio nuts. Food Microbiol..

